# The Enforcement of Moral Boundaries Promotes Cooperation and Prosocial Behavior in Groups

**DOI:** 10.1038/srep42844

**Published:** 2017-02-17

**Authors:** Brent Simpson, Robb Willer, Ashley Harrell

**Affiliations:** 1 Department of Sociology, University of South Carolina, Columbia, SC 29208, USA; 2 Department of Sociology, Stanford University, 450 Serra Mall, Bldg. 120, Stanford, CA, 94305, USA

## Abstract

The threat of free-riding makes the marshalling of cooperation from group members a fundamental challenge of social life. Where classical social science theory saw the enforcement of moral boundaries as a critical way by which group members regulate one another’s self-interest and build cooperation, moral judgments have most often been studied as processes internal to individuals. Here we investigate how the interpersonal expression of positive and negative moral judgments encourages cooperation in groups and prosocial behavior between group members. In a laboratory experiment, groups whose members could make moral judgments achieved greater cooperation than groups with no capacity to sanction, levels comparable to those of groups featuring costly material sanctions. In addition, members of moral judgment groups subsequently showed more interpersonal trust, trustworthiness, and generosity than all other groups. These findings extend prior work on peer enforcement, highlighting how the enforcement of moral boundaries offers an efficient solution to cooperation problems and promotes prosocial behavior between group members.

The “cooperation problem” refers to the challenge groups face in motivating their members to set aside narrow self-interest to make costly contributions to collective efforts^1^,^2^,^3^,^4^,^5^. Following early sociological claims^6^,^7^ and anthropological accounts^8^,^9^, moral psychology views morality as critical to social order, but overwhelmingly moral reasoning and judgment are studied as internal psychological processes, as noted in a review of the literature^10^. As a consequence, we know comparatively little about the communication of interpersonal moral judgments to others and how expressed moral judgments may impact individual behavior and group processes^11^,^12^.

Here we show that the communication of interpersonal moral judgments is an important means through which groups solve cooperation problems. Because individuals deeply value moral praise and fear moral condemnation, the prospect of interpersonal moral judgments can serve as a powerful tool for maintaining social order and motivating actions that benefit groups or group members. This view contrasts with dominant explanations of the impact of peer sanctions, which focus on how material sanctions lead would-be free-riders to contribute their share to group efforts^13^. In these accounts, sanctions from fellow group members matter because they bring cooperation in line with individual self-interest, and moral praise or condemnation is largely “cheap talk” that does not affect contributions. Accordingly, the literature operationalizes sanctions as monetary fines or rewards deployed at a material cost by other group members^14^. A few prior studies, however, have looked at costless, or non-monetary, expressions of approval or liking, and these have yielded mixed evidence for their effectiveness in promoting contributions to collective action^15^,^16^,^17^. While suggestive that non-monetary sanctions can impact cooperative behavior, these studies did not allow group members to communicate *moral* judgments of one another’s behaviors. We argue below that moral judgments are likely to promote cooperation. Just as importantly, prior work has not yet studied other key consequences of monetary sanctions versus social or moral judgments, though these may have very different downstream effects. Here we show that, unlike material sanctions, moral judgments do not result in cycles of recrimination and tend to “crowd in” interpersonal trust, trustworthiness, and generosity.

## Theoretical Background

Individuals form internal moral judgments of others’ actions spontaneously^18^, and frequently communicate them to others^19^,^20^,^21^. Further, people are both intrinsically and extrinsically motivated by others’ moral judgments. For instance, individuals value maintaining views of themselves as moral individuals^22^,^23^. In addition, individuals are highly motivated to maintain a reputation as a moral person in the eyes of others^24^,^25^, a functional motivation given that morality is the trait observers care most about when forming impressions of others^26^,^27^,^28^. Indeed, a large body of interdisciplinary work underscores the important role moral and prosocial reputations play in promoting cooperation and other forms of prosocial behavior^29^,^30^. Finally, prior work on dyadic interactions shows that those who morally judge unfair behaviors subsequently perceive themselves as more moral and act more trustworthy. Their trustworthiness is also anticipated by observers who prefer them as interaction partners in situations involving risk and uncertainty^11^,^12^. We therefore expect that the capacity for group members to make moral judgments about one another will promote cooperation in groups.

Given the centrality of morality and reputations to social life, it is perhaps unsurprising that ethnographic findings show that moral judgments are a common means through which groups facing cooperation problems coordinate expectations of acceptable behavior^8^,^9^. Conversely, use of material punishments, where an individual or group pays a cost to exact a larger cost on a peer is less common^14^,^31^. Studies of vigilantism show that citizens may direct violence against law breakers when institutions are perceived as weak or illegitimate. Such exceptions notwithstanding, where material sanctions are present, they are generally administered by centralized agents or other institutions, not peers^32^,^33^.

Although there is no theoretical basis for predicting differences in cooperation rates between groups whose members can make moral judgments of one another versus groups who can materially sanction one another, there are clear theoretical reasons to expect divergent effects of moral judgments and material sanctions with respect to other group dynamics. For instance, while past work finds that material sanctions can beget cycles of retribution, disassociating sanctions and contribution decisions^34^,^35^, we expect these cycles will be less common in the case of moral judgments. Where those deploying costly material punishments are often assumed to be antisocially motivated^31^,^36^,^37^, past research finds the motivations of moral judges are typically assumed to be prosocial^11^,^12^. Given that moral judgments are generally presumed to be based in sincere prosocial motivations, we expect that targets of moral judgments will be less apt to respond with recrimination, compared with targets of material punishment.

More importantly, we expect that the capacity for groups to communicate interpersonal moral judgments will promote interpersonal trust, trustworthiness, and generosity among group members in subsequent interactions where the capacity has been removed. As noted above, material sanctions tend to increase contributions in the short run. Yet past research suggests that these effects are limited to situations where sanctions are continually threatened and that, once removed, sanctions inhibit trust and prosociality^38^,^39^. Material sanctions can inhibit the development of trust by prompting uncertainty regarding the motives underlying others actions^4^. Where the threat of sanctions is present, individuals do not know if their fellow group members behaved prosocially because of the threat of punishment or from a sincere desire to benefit the group. On the other hand, moral judges are trusted more than others, behave in more trustworthy ways, and perceive themselves to be more trustworthy^11^,^12^. We thus expect that moral judgments will promote interpersonal generosity, trust, and trustworthiness toward fellow group members.

Summing up, we test two core hypotheses. Hypothesis 1 states that, like material sanctions, the capacity of group members to make moral judgments of one another will increase contributions, relative to control groups. Hypothesis 1 may be supported via a *deterrence process*, a *learning process,* or both. That is, the mere presence of moral judgments or material sanctions may lead to higher levels of cooperation, even before they are administered (*deterrence*). Or recipients of judgments or sanctions may increase their contributions after being judged or sanctioned (*learning*).

Hypothesis 2 states that, compared to groups with material sanctions and control groups, those whose members can make moral judgments of one another will behave more prosocially toward one another (higher levels of generosity, trust, and trustworthiness) in subsequent interactions.

### Relation to Prior Work

Although no prior work has addressed the consequences of interpersonal moral judgments in collective action contexts, a few studies have shown that anticipating approval or disapproval from a potential recipient leads to increased giving in a dictator game^40^,^41^. Several other studies have addressed abstract expressions of approval or liking but found somewhat mixed conclusions regarding their effectiveness. Two studies that allowed participants to assign costless disapproval points found that they initially led to increased contributions, but these gains were short-lived, relative to a monetary punishment condition^16^,^17^. Another study found that allowing group members to send “smiley” or “frowny” faces did not result in higher levels of contributions relative to control groups^15^.

While results from these prior studies of collective action are mixed, we have argued that the ability to communicate *moral* approval or disapproval, in particular, will effectively promote contributions in collective action contexts. That is, we cannot know from prior studies whether responses to abstract expressions of approval or disapproval were driven by moral concerns. One goal of a pilot study we conducted (see Supplementary Information) was to test whether the capacity for individuals to express judgments of one another’s moral standing is especially likely to promote cooperation, over and above expressions of approval or disapproval on another core dimension along which people evaluate themselves and others. Specifically, we compared the effects of morality judgments to judgments of competence because competence is the other fundamental, socially valued, yet non-moral, dimension of perception and social judgment^26^,^28^,^42^. In line with Hypothesis 1, results of the pilot experiment showed that groups whose members have the capacity to make interpersonal judgments about one another made greater monetary contributions to a shared resource pool that benefited all group members, and also reported stronger feelings of group solidarity, but did so only when those judgments occur along the moral dimension.

Just as importantly, we go beyond prior work by studying advantageous downstream effects of moral judgments. Prior work has not addressed whether non-material sanctions mitigate the problems of material sanctioning systems discussed above, namely whether or not they lead to cycles of recrimination, and whether they promote interpersonal generosity, trust and trustworthiness between group members. This is an important omission, given prior demonstrations that the efficacy of material sanctions is limited to situations where they are continually threatened and that, once removed, they may actually inhibit trust and prosociality^38^,^39^. One of our key goals is to address whether the ability of group members to make moral judgments of one another will promote less conflictual and more harmonious interactions within groups, as predicted by Hypothesis 2.

Also relevant is prior work demonstrating that communication promotes cooperation in social dilemmas^43^,^44^,^45^. While there have been various accounts of the mechanism underlying the positive effects of communication, a standard explanation is that it creates the conditions for promise-making (and keeping), facilitating the emergence of prosocial norms of cooperation^46^,^47^. Consistent with this account, an extensive review of the literature^47^ showed that positive effects of communication are primarily limited to face-to-face communication or video conferencing. The benefits of mere communication do not extend to other media, such as text or computer-mediated messages, as they do not create the conditions for group members to credibly communicate promises to cooperate. Importantly, the communication *medium* we use to test our moral judgments predictions is the type that prior work shows is insufficient for mere communication effects on levels of cooperation. Nonetheless, Hypothesis 1 predicts that the capacity to make moral judgments of fellow group members will increase contributions relative to control groups, for the reasons we outlined above.

## Results

Our experiment addresses the impact of interpersonal moral judgments and material sanctions on both public goods contributions and downstream prosocial behaviors in groups of anonymous strangers. Anonymous groups provide a strong test of whether moral judgments can promote cooperation since others’ moral judgments cannot have lasting effects on moral reputations, as would be the case in groups where members have ongoing relationships.

As detailed in the Methods section, members of interpersonal moral judgment (MJ) groups could make positive or negative moral judgments of one another after each round of public goods dilemma (PGD) contributions, by rating each other’s behavior on a scale of “very immoral” to “very moral.” Members of material sanctions (MS) groups could materially reward or punish one another after each round. To help ensure that comparisons between moral judgments and material sanctions did not depend on any particular operationalization of material sanctions, we ran a “private” and “public” MS condition (see *Methods* and Supplementary Information). As in the MJ condition, participants in the public MS condition were given full information about the rewards and punishments sent and received by all others in the group. Those in the private MS condition could see how their earnings were modified as a result of sanctions but did not know which group member sent them. Nor could they view the sanctions received by anyone else in their group. As detailed in the Supplementary Information, we did not find any differences between the private and public MS conditions, consistent with the view that monetary sanctions have their effects via the transformation of material incentives (versus the public expression of moral approval or disapproval). Analyses reported in the Supplementary Information show that, for all tested outcome variables, the private and public MS conditions did not differ from one another. Our primary analyses are thus based on combining the two MS conditions. We compare contributions in MJ and MS groups to those in a control condition with no judgments or sanctions.

**Figure 1:**
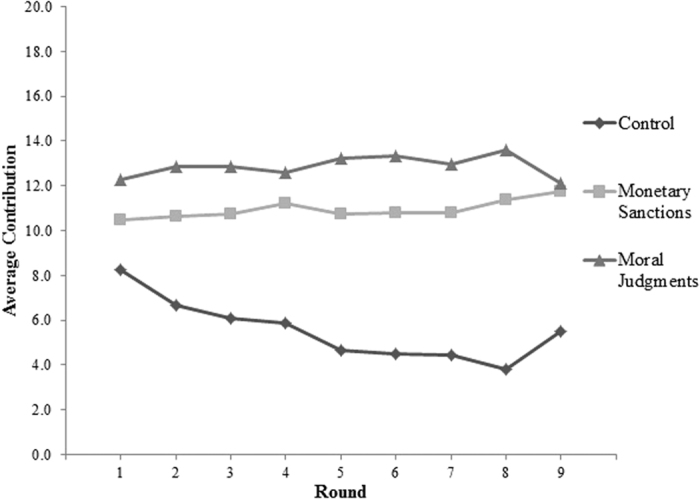
Contributions by Condition and Round.

Participants knew ahead of time that they would be able to send MJs or MSs to others. Thus, we can assess whether moral judgments and material sanctions deterred uncooperative behavior in round 1, even before judgments or sanctions were used. Consistent with Hypothesis 1, Fig. 1 and Table S5 (Supplementary Information) show that even in the first round, before any sanctions or judgments were sent, participants in MJ and MS conditions contributed 49% and 27%, respectively, more than those in control groups (*Bs* = 4.02 and 2.22, *d*s = 0.60 and 0.33, *p*s = 0.01 and 0.09, respectively). First round contributions in MJ groups were not significantly different from MS groups (*B* = 1.80, *d* = 0.28, *p* = 0.14). These first-round contributions reveal a deterrence process, such that members of MJ groups responded to the mere prospect of being morally judged by strangers by increasing their contributions to the public good. These findings are consistent with prior research on the effects of anticipated verbal feedback on prosocial behavior^40^,^41^.

Control groups showed the characteristic decline in contributions across rounds, but cooperation remained significantly higher in MJ and MS groups across the remaining eight rounds of the PGD, as shown in Fig. 1 and Table S5 (*B*s = 7.75 and 5.81, *d*s = 1.12 and 0.83 respectively, *p*s < 0.001). By the final round, participants in the MJ and MS conditions were contributing over twice as much as those in the control conditions. Moreover, cooperation in MJ groups remained non-significantly higher than in MS groups for the remaining eight rounds (*B* = 1.94, *d* = 0.28, *p* = 0.24). Thus, supporting Hypothesis 1, participants in MJ groups contributed significantly more than those in control groups throughout the study, giving at levels comparable to that of participants in MS groups.

**Figure 2:**
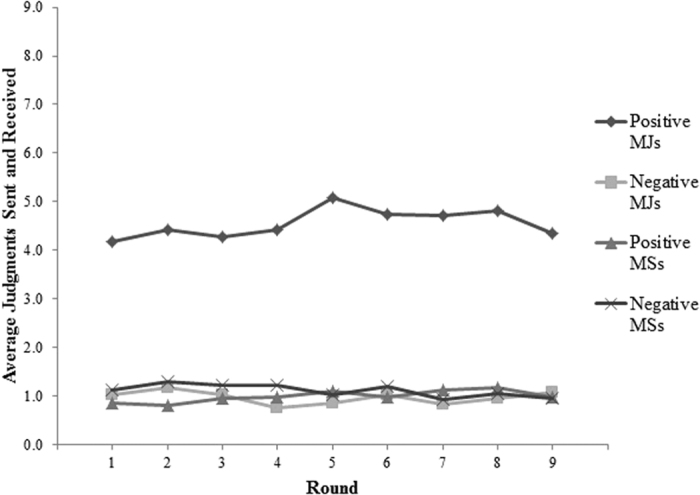
Average Judgments/Sanctions Sent and Received by Valence, Condition and Round. Note: Figure displays the average sum of (absolute value) negative and positive moral judgments (MJs) and material sanctions (MSs) that participants sent and received. (Note that, at the group level, judgments/sanctions sent equal judgments/sanctions received.) Possible values ranged from 0 [the group sent/received no negative (positive) MJs (MSs)] to 9 [the group sent/received the maximum number of negative (positive) MJs (Ms)].

We averaged the three judgments or sanctions a participant received from the three others into a single “average judgment” or “average sanction” score that could range from −3 to 3. Figure 2 displays patterns of moral judgments and material sanctions over time. Participants in MS groups deployed costly sanctions 36% of the time, and those sanctions were equally likely to be positive as negative. Moral judgments were more common, with participants in MJ groups making judgments of one another on 74% of opportunities to do so, with positive moral judgments being more common (60%) than negative judgments (14%).

We now examine how contributions impacted the average judgment or sanction a participant received from the three others in a given round. Because participants in the Control condition did not send or receive judgments, they are omitted from these analyses. These analyses, reported in Table S6, show significant main effects of both condition and contribution, and a significant interaction of condition and contribution. The main effect of condition suggests that participants who gave nothing received more negative MJs than MSs (*B* = −0.53, *p* < 0.05). The main effect of contribution (*B* = 0.08, *p* < 0.001) suggests that participants who contributed more received more positive judgments or sanctions. The interaction (*B* = 0.13, *p* < 0.001) suggests that the relationship between contributions and average judgment/sanction received was particularly strong in the MJ condition. Thus, we observed a strong, positive relationship between individuals’ contributions and the judgments and sanctions they subsequently received, and this relationship was stronger in MJ than in MS groups.

Beyond the impact of contributions in the prior round on judgments and sanctions received from others, we also tested whether judgments and sanctions received impacted the recipient’s subsequent contributions. The model, given in Table S7, controlled for the recipient’s contribution and judgments sent in the previous round; it also controlled for the group’s average contribution in the previous round. Because Control participants did not make judgments, and because participants in the Private MS condition could not see group-level judgments, participants from those study sessions were omitted from this analysis.

**Figure 3:**
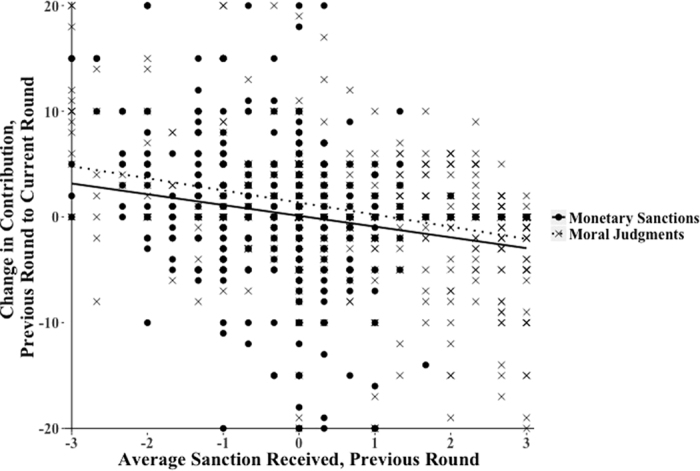
The Effects of Sanctions and Judgments Received on Change in Public Good Contributions. Note: Figure displays all observed data points for participants in the moral judgments condition and the public material sanctions condition. The x-axis gives average sanction or judgment received from the other group members following round *t* contributions; the *y*-axis gives change in contribution in the subsequent round.

The relationship between judgments or sanctions received and contributions was negative (*B* = −0.62, *p* = 0.02), such that the more negative the average judgment or sanction received, the more participants tended to increase their contributions, relative to the prior round’s contribution level. Figure 3 gives a scatterplot of the relationship between sanctions or judgments received and change in contributions in the subsequent round. The figure shows that there was a tendency for negative sanctions and judgments to be followed by increased contributions and positive sanctions and judgments to be followed by decreased contributions. That participants altered their contributions in response to moral judgments and monetary sanctions is consistent with a learning process.

As shown in Table S8, the efficiency losses from material sanctions^48^,^49^ led to lower overall earnings in MS compared to MJ groups. Factoring in the costs and benefits associated with sending and receiving material sanctions, MJ group members earned 15% more than MS group members (*B* = 4.19, *d* = 0.47, *p* = 0.07). MJ groups members also earned 28% more than Control group members (*B* = 7.33, *d* = 1.06, *p* = 0.01). Earnings in MS groups did not differ significantly from those in Control groups (*B* = 3.14, *d* = 0.35, *p* = 0.19).

Besides the efficiency losses incurred by sending and receiving material sanctions, another critical drawback of material sanctions is that they can invite retaliation from recipients^34^,^35^. To examine retaliation and reciprocation of judgments and sanctions, we considered negative (Model 1) and positive (Model 2) sanctions and judgments separately. The models, given in Table S9, show how the sanctions or judgments a participant sent to a given alter in the previous round impacted sanctions or judgments received from that same alter in the current round, net of a range of control variables. (Only participants in the public MS condition were able to see who had punished or rewarded them and how much. We therefore omitted the private MS groups from these analyses).

Model 1 shows no main effect of negative judgments/sanctions previously sent to alter on sanctions received from that alter in the next round. However, there was a significant interaction. Specifically, in line with prior studies, targets of material punishments frequently retaliated by punishing those who punished them in the previous round (*B* = 0.18, *p* < 0.001). However, we found no such tendency among those in MJ conditions who were morally condemned (*B* = 0.02, *p* = 0.37). This pattern of retaliation is consistent with the idea that material punishments are often perceived as antisocial by recipients^31^,^36^, whereas the motivations underlying moral judgments are more likely to be viewed as prosocial^11^,^12^.

As shown in Model 2, we observed similar differences for positive interactions. But in this case, participants reciprocated positive moral judgments (*B* = 0.08, *p* < 0.05), but were more likely to reciprocate material rewards (*B* = 0.16, *p* < 0.05).

After nine rounds of the PGD, participants completed a measure of solidarity (Cronbach’s alpha = 0.83). As shown in Table S10, both MJ and MS groups had higher solidarity than control groups (*Bs* = 1.13 and 1.16 respectively, both *d*s = 0.80, *p*s < 0.01). There was no significant difference between MJ and MS groups (*B* = 0.02, *d* = 0.02, *p* = 0.95).

Each group made decisions in an additional, single-round PGD, where they were told that the opportunities to judge or sanction others would no longer be present. Table S11 (Model 1) shows that participants in MJ groups contributed 83% more in the one-shot PGD, and participants in MS groups contributed 56% more, than those in the control group (*Bs* = 4.91 and 3.34, *d*s = 0.63 and 0.44, *p*s = 0.02 and 0.07). Contributions in MJ groups were non-significantly higher than MS groups (*B* = 1.57, *d* = 0.21, *p* = 0.37).

**Figure 4:**
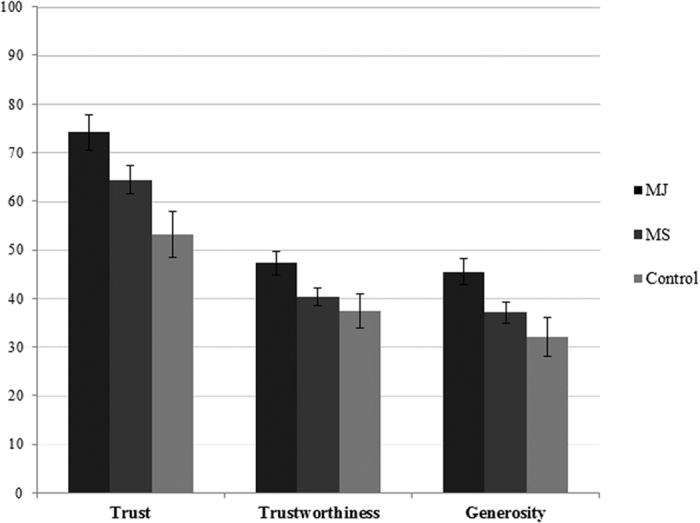
Trust, Trustworthiness and Generosity by Condition. Note: Figure displays the percentage of the endowment entrusted (as Trustor in TG), returned (as Trustee in TG), and given (in DG), respectively, in each of the three experimental conditions. Error bars give the standard error of the mean. For each measure, N = 48, 60 and 104 participants in the Control, MJ, and MS conditions respectively.

The remaining three models of Table S11 report results for our three measures of prosociality in dyadic interactions with fellow group members and Fig. 4 displays means for these dyadic measures across experimental conditions. We expected that moral judgments would be more likely to promote generosity, trust, and trustworthiness than material sanctions. As shown in Model 2, participants in MJ groups gave more to their partners in the dictator game than either those in Control groups (*B* = 1.34, *d* = 0.55, *p* = 0.01) or MS groups (*B* = 0.84, *d* = 0.39, *p* = 0.05). Control and MS group members’ giving did not differ significantly (*B* = −0.50, *d* = 0.20, *p* = 0.26).

Similarly, Model 3 shows that trust was higher among MJ group members than members of Control (*B* = 2.10, *d* = 0.69, *p* < 0.01) or MS groups (*B* = 0.97, *d* = 0.34, *p* = 0.09). MS group members were also more trusting than Control groups (*B* = 1.13, *d* = 0.36, *p* = 0.07). Finally, Model 4 shows that higher amounts were returned by Trustees who participated in an MJ group, compared to those from Control groups (*B* = 9.92, *d* = 0.45, *p* < 0.01) or MS groups (*B* = 6.92, *d* = 0.37, *p* = 0.03). The latter two conditions were not significantly different (*B* = 3.00, *d* = 0.14, *p* = 0.37). Thus participants who had been in groups in which members had the opportunity to morally judge one another were more generous, trusting, and trustworthy in subsequent interactions with fellow group members than those from groups whose members could materially sanction one another or control groups. These results provide strong and consistent support for Hypothesis 2 and, more generally, show that moral judgments lead to lower levels of retaliation, and higher levels of generosity, trust, and trustworthiness compared to material sanctions.

## Discussion

Recent research has yielded critical insights into how people form internal moral judgments, but has less often studied the communication of moral judgments to fellow group members, and how expressed judgements may impact individuals and groups. This is a surprising omission given research establishing the powerful role of reputational processes in fostering cooperation and prosocial behavior^4^,^5^,^21^,^30^. Our findings show that groups whose members could express public, moral judgments of one another marshalled greater costly contributions to group efforts and showed higher solidarity than groups that could not express such judgments, levels of contribution and solidarity that were comparable to groups in which members could deploy material sanctions (Hypothesis 1). Further, we found that the effect of moral judgments on contributions – like material sanctions – operated via both deterrence and learning processes. But unlike material sanctions, moral judgments did not result in cycles of retaliation. But unlike material sanctions, moral judgments did not result in cycles of retaliation. Finally, in subsequent dyadic interactions, we found that members of moral judgment groups were more generous, trusting, and trustworthy than members of either groups that could not express judgments, or groups that featured material sanctions (Hypothesis 2). Thus, moral judgments were an effective and efficient mechanism for fostering cooperation, solidarity, generosity, trust, and trustworthiness.

One way of thinking about our findings centers on the ambiguity in the literature about whether monetary sanctions impact cooperation via their impact on material outcomes, or whether monetary sanctions are simply convenient proxies for the social and affective costs and benefits of sanctions^14^. For example, in field settings, sanctioners face risks of retaliation, recipients of negative sanctions may feel shame and loss of social standing, and recipients of positive sanctions may experience pride and enhanced prestige. Viewed from this perspective, our research disposes of material sanctions as rough proxies for social and affective costs and benefits to more directly study the properties of one form of nonmaterial, social sanction. In so doing, we find important differences between moral and material sanctions (with respect to retaliation and downstream trust, trustworthiness, and generosity), suggesting that the use of the latter as proxy for the former is imprecise and risks misunderstanding the true dynamics of social sanctioning regimes and their influence on cooperation in groups.

More generally, our findings speak to a tension between two theoretical traditions, emphasizing very different foundations of productive collective action and solidarity in groups. Where interest-based theories assert that prosocial actions result from the alignment of group members’ material interests with collective goals, morality-based theories emphasize interpersonal moral judgments and the development of moral consensus that contributions to group efforts are proper and just. These traditions cast very different roles for peer sanctioning, as either tools for modifying the material payoff structures of actors, or mechanisms of social judgment that clarify and enforce moral boundaries by praising contributors and shaming non-contributors.

Of these traditions, material sanctioning has emerged in the past decade as the most prominent solution in the literature on collective action. And these studies have yielded a number of critical insights into the evolution of sanctioning systems. But here we find that moral judgments offer an equally effective, costless mechanism for promoting group productivity and harmony, one that members spontaneously deployed given the opportunity. Moral judgments not only allowed for the efficient promotion of collective action; they also built positive sentiments between group members, as revealed in their subsequent cooperative interpersonal encounters. These findings suggest that the motivation to see ourselves, and be seen by others, as moral actors is every bit as strong as the drive to maximize material profit. As a result, the clarification and enforcement of moral boundaries, and the tacit coordination of moral order, offers a basis for harmonious group living.

## Methods

Participants were recruited from the general student population at a large public University and scheduled in groups of four. Upon arrival to the research laboratory, each participant was escorted to a private computer terminal where she completed the consent process and began the instructions. All instructions and interactions took place anonymously via computers, using Z-Tree software, version 3.3.12 50. Based on sample sizes in the pilot experiment (see Supplementary Information) and in related work^13^,^19^ we ran 54 four-person groups, randomly assigned by the computer to one of four conditions: *interpersonal moral judgments* (MJ, N_groups_ = 15), two variations on a material sanctions condition (MS, N_groups_ = 13 each), and a control condition (N_groups_ = 13) with no judgments or sanctions.

Participants played nine rounds of the public goods dilemma (PGD). They were not told in advance how many rounds the PDG would last. In each round, each of the four group members was given an endowment of 20 monetary units (MUs). Each decided how many, if any, of their MUs to contribute to the public good. Contributions to the public good fund were doubled and distributed equally among all group members, regardless of how much they contributed (see Supplementary Information for the full text of all study instructions). Thus, withholding contributions maximizes an individual’s earnings, but overall group earnings are maximized when all group members contribute to the public good. After each round of contributions, participants could see the contributions and earnings of all group members for that round. Thereafter those in the MJ and MS conditions had the opportunity to express moral judgments or to administer monetary sanctions.

To increase comparability between MJ and MS conditions, those in the MJ condition could make interpersonal moral judgments of one another using a single item ranging from −3 (very immoral) to + 3 (very moral), where 0 indicated “no judgment.” Participants were informed in the study instructions of the type of judgments they would make about one another before the first round. Thus, if the mere prospect of being morally judged encourages cooperation, we should observe an effect of MJs on contributions in the first round, before any judgments are actually made.

Those in the two material sanctions conditions indicated whether they wished to deduct from (punish) or add to (reward) each of their group members’ earnings using a scale of −3 to 3, where −3 indicated they wished to spend three of their points to deduct nine points from the other’s earnings, +3 indicated they wished to spend three of their points to add nine points to the other’s earnings, and 0 indicated they did not wish to make any changes to the other’s earnings for that round. Thus, following prior studies^13^,^21^,^33^, each monetary sanction cost one MU and either benefited or cost the recipient three MUs. (For each round, each participant could spend up to three MUs to punish or reward each other group member.) A meta-analysis concluded that the impact of monetary sanctions on cooperation did not depend on the “cost to fine” ratio, meaning that sanctions had similar effects on cooperation whether a punisher paid one MU to reduce another’s earnings by one, two, three, or four MUs^51^. Thus, while at the extremes we could well imagine a calibration of material sanctions that would have larger effects than MJs (e.g., a material sanction that was very low cost for the sanctioner, but extremely costly to the recipient), results from the meta-analysis suggest that our results should be robust to the range of magnitudes of monetary sanctions that have been investigated in previous research.

Results from the same meta-analysis also showed that (positive or negative) sanctions that are costly to send are more effective at promoting cooperation than those that are costless^51^. Thus making material sanctions costly and moral judgments costless provides a conservative test of our claims about the power of moral judgments versus material sanctions to promote cooperation and is consistent with work on non-material sanctions discussed earlier^16^,^17^.

As noted above, we conducted two different MS conditions, in order to ensure that our comparisons were robust across different operationalizations of material sanctions. As shown in the Supplementary Information, we did not find any differences between the “private” and “public” MS conditions. Our analyses are thus based on combining the two MS conditions.

To assess the downstream effects of moral judgments and material sanctions on solidarity and intrinsic motivation, after nine rounds of the PGD, participants completed a measure of solidarity^24^. Participants then made decisions in a final, one-shot, PGD with no judgments or sanctions. Finally, they made decisions in a series of one-shot dyadic interactions measuring generosity, trust, and trustworthiness. For each of these decisions, each participant was paired with a unique member from their group.

We used a standard behavioral measure of generosity, the Dictator Game^52^. The game is a situation of unilateral dependence where one person, the “Dictator,” is given an endowment of 10 MUs and must decide how many, from 0 to 10, to transfer to a Recipient. (For all these decisions, actual experimental instructions used benign terms like “sender,” “receiver,” etc.). The Recipient’s outcome for the interaction depends solely on the Dictator’s decision and generosity is costly for the Dictator.

The trust and trustworthiness measures were based on the standard Trust Dilemma^21^,^39^. For the trust measure, participants occupied the role of *Trustor*. The Trustor was allocated 10 MUs and was told that he or she could send any amount of the 10 MUs to the Trustee. Any amount sent to the Trustee would be tripled. (For instance, if the Trustor sent all 10 MUs, the investment would yield 30 MUs). Trustees decided what percent of the tripled amount he or she would keep to himself versus return to the Trustor. Participants took part in one Trust Dilemma as Trustor and another as Trustee.

Finally, participants were paid for their earnings, which ranged from 10 to 15 dollars, with an average of $13.09. The study took an hour to complete. There was no deception. The experimental procedures were approved by the Institutional Review Board at the University of South Carolina. Informed consent was obtained from all study participants and the study was carried out in accordance with the approved guidelines and procedures.

Because the data consisted of round-by-round observations nested within participants, which were nested within groups, our analyses are based on multilevel models (see Supplementary Information for details on all models).

### Availability of Data and Syntax

Data from both experiments and syntax for all analyses are available here: https://www.dropbox.com/sh/hwdkbkf0k5gd7v2/AABAW38-yCWDbPtcBmKm0Wla?dl=0.

## Additional Information

**How to cite this article**: Simpson, B. *et al*. The Enforcement of Moral Boundaries Promotes Cooperation and Prosocial Behavior in Groups. *Sci. Rep.* **7**, 42844; doi: 10.1038/srep42844 (2017).

**Publisher's note:** Springer Nature remains neutral with regard to jurisdictional claims in published maps and institutional affiliations.

## Supplementary Material

Supplementary Information
