# Supplementary material for: The Enforcement of Moral Boundaries Promotes Cooperation and Prosocial Behavior in Groups
Source: Sci Rep. 2017 Feb 17;7:42844. doi: 10.1038/srep42844 (PMC5314330; doi:10.1038/srep42844)
Supplement: Supplementary Information [file srep42844-s1.pdf]

Supplementary Information

The Enforcement of Moral Boundaries Promotes Cooperation and Prosocial Behavior in Groups

Authors: Brent Simpson,[1*] Robb Willer,[2] and Ashley Harrell[1]

1 Department of Sociology, University of South Carolina, Columbia, SC 29208, USA.

2 Department of Sociology, Stanford University, 450 Serra Mall, Bldg. 120, Stanford, CA, 94305, USA.

Corresponding Author:
Brent Simpson
Department of Sociology
University of South Carolina
Columbia, SC 29208
bts@sc.edu
(803) 315-3259

Pilot Experiment

The primary aim of the pilot experiment was to provide an initial test of whether the enforcement of moral boundaries via interpersonal moral judgments promotes cooperation in groups of anonymous strangers. We also wanted to ensure that our predicted effects were limited to moral judgments in particular. Thus, in addition to a control condition, we compared groups who could make moral judgments to groups who could make *competence judgments* because competence is the other fundamental, socially valued, yet nonmoral, dimension of perception and judgment[1,2].

Participants were recruited from the general student population at a large public University and scheduled in groups of four. Upon arrival to the research laboratory, each participant was escorted to a private computer terminal where she completed the consent process and began the instructions. All instructions and interactions took place anonymously via computers, using Z-Tree software, version 3.3.12 [3]. Our analyses are based on 34 four-person groups.[1] These groups were randomly assigned by the computer to one of three conditions: interpersonal moral judgments (MJ), interpersonal competence judgments (CJ) and control.

In each round, each of the four group members was given an endowment of 20 monetary units (MUs). Each decided how many, if any, of their MUs to contribute to the public good. Contributions to the public good fund were doubled and distributed equally among all group members, regardless of how much they contributed. After each contribution phase, the computer displayed each group member's contributions and earnings for that round. Thereafter, participants in the two judgment conditions could make (moral or competence) judgments of each other group member. Those in the MJ condition indicated how *moral* and *fair* each other group member was on a scale from 1 to 7 where, e.g., 1 = very unfair, 4 = neither fair nor unfair, and 7 = very fair; similarly, 1 = very immoral, 4 = neither immoral or moral, and 7 = very moral. Those in the CJ condition rated how *competent* and *capable* each other group member was on two separate 7-point scales. Although participants were told in advance that they would be rating other group members, for the pilot experiment, they did not know the content of those ratings prior to the first round of contributions. Participants in the control condition did not make judgments, as in the standard public goods design. (See the *Full Text of Study Materials* section below for all study materials.)

After participants completed the judgments phase of each round, they viewed a table showing how each group member judged them, and how they (and others) judged each other group member. Rather than displaying each group members' responses to both judgment questions, the table reported a single judgment score from each participant to each participant, i.e., the average of the two judgment questions. Thereafter, participants moved on to the next round, where they again decided how much, if any, of a new 20 MU endowment to contribute to the group. The entire process was repeated for a total of nine rounds, which is typical for related studies, which generally range anywhere from four to 15 rounds[4-6]. Participants were not aware of how many rounds the study would last. All earnings from each round were added to the total

[1] One group in the competence judgments (CJ) condition was omitted from analyses because one participant in the group reported suspicion that they were actually interacting with others during the study, and another participant reported substantial confusion about the study instructions. Thus, 34 four-person groups were included in the analyses reported here—13 groups in the moral judgments (MJ) condition, 10 in CJ, and 11 in Control.

earnings. Following nine rounds of the public goods dilemma, participants completed an index of solidarity [7]. Each session lasted just under an hour and payments ranged from $10 to $15 (average $12.54). There was no deception. The experimental procedures were approved by the Institutional Review Board at the University of South Carolina. Informed consent was obtained from all study participants and the study was carried out in accordance with the approved guidelines and procedures.

Results of Pilot Experiment

We compare patterns of contributions in the moral and competence judgments conditions to those in a control condition, where people could not submit any judgments about one another's contributions. Data in both the pilot study and the study reported in the main text consisted of round-by-round observations nested within participants, which were in turn nested within groups. Because nested data violates several of the assumptions required for traditional regression (including independence of cases and homogeneity of variance), all analyses reported here are based on multilevel models.

As noted above, although participants in the pilot study knew before the first round that they would be making judgments of one another following their contributions, they did not know the type or content until the ratings were solicited following first round contributions. Figure S1 shows that contribution differences emerged in the second round, immediately after participants knew what types of judgments they and other group members would be making. Results of statistical analyses reported in Table S1 show that across the ensuing eight rounds, participants in MJ groups contributed significantly more than those in either CJ or control groups (*B*s= 5.05 and 5.34 and *d*s= .81 and .75, respectively, *p*s < .01), which did not differ from each other (B = .29, d = .04, p = .88). Thus, while control and CJ groups show the decline in cooperation over time that is typical in experimental public goods settings [8,9], contributions in the MJ groups remained high across all interaction rounds. These results support our prediction that interpersonal moral judgments will promote cooperation.

After nine rounds of the public goods dilemma, participants completed the survey battery measuring feelings of group solidarity. Responses to the three questions were highly reliable (Cronbach's alpha = .85). We therefore averaged responses to the three questions to create one average solidarity rating (overall M = 3.39, SD = 1.49). The results are reported in Table S2. In line with our behavioral results, we found higher levels of solidarity among participants in MJ groups (M = 4.03) than in either CJ (M = 3.25) or Control (M = 2.77) groups (B = .78 and 1.27, d = .52 and .97, p < .05 and p < .01, respectively). There was no significant difference in solidarity ratings of CJ and control groups (B = .48, d = .34, p = .21).

Discussion of Pilot Experiment and Motivation for Main Study

The pilot experiment shows that moral judgments promote cooperation, but it does not tell us why. The higher cooperation in MJ groups beginning in the second round is consistent with at least two different, non-competing, mechanisms. A deterrence effect could have led participants to increase their contributions, relative to those in control and CJ groups, upon being informed of the type of judgments others would be making of them. These findings could also result from a learning effect, whereby those receiving more negative moral judgments subsequently increased their contributions to bring them in line with judges' expectations. Because participants in the pilot experiment did not know what form of judgments others would make of them until after the

first round of contributions were submitted, we could not assess whether the higher contributions in MJ groups were driven by deterrence, learning, or both. Our primary experiment, reported in the main text, allows us to address this gap.

Comparison of Public and Private Material Sanctions Conditions for Primary Experiment

As described in the main text, we conducted two different variations of the material sanctions condition for the primary experiment: Public MS (N_{groups} = 13) and Private MS (N_{groups} = 13). All analyses reported in the main text are based on a single MS condition (N_{groups} = 26) that combines these two variations. Here we justify aggregating the two treatments by showing that they did not differ from each other for any comparisons. Table S3 shows that Public MS and Private MS groups did not differ in their contributions, sanctions received, or earnings during the public good task. Table S4 shows that Public MS and Private MS groups did not differ in any of the post-study measures reported above, including solidarity, contribution in the one-shot PGD, trust, trustworthiness, and generosity.

Table S1. Contributions to the Public Good, Pilot Experiment

	Model 1: Contributions, Round 1		Model 2: Contributions, Rounds 2-9	
	Estimate	S.E.	Estimate	S.E.
Intercept	9.08***	.90	11.00***	1.15
Control	-.01	1.33	-5.05**	1.70
CJ	-.43	1.37	-5.34**	1.74
	$N_{\text{observations (groups)}}$= 136 (34)		$N_{\text{observations (groups)}}$= 1088 (34)	

+ *p* < .10, * *p* < .05, ** *p* < .01, *** *p* < .001

Note. Two-level (Model 1) and three-level (Model 2) multilevel regression models. Two-tailed tests. The dependent variable is contribution in round 1 for Model 1 and contribution in rounds 2 through 9 for Model 2; possible values range from 0 to 20. MJ is the reference category. We also compared the Control and CJ conditions to each other, by changing the reference category to CJ. For both Models 1 and 2, the Control-CJ comparison did not differ (*p* = .77 and *p* = .88, respectively).

Table S2. Perceived Solidarity, Pilot Experiment

	Average Solidarity Rating	
	Estimate	S.E.
Intercept	4.03***	.24
Control	-1.27**	.35
CJ	-.78*	.36
	$N_{observations\ (groups)}$= 136 (34)	

+ $p < .10$, * $p < .05$, ** $p < .01$, *** $p < .001$

Note. Two-level regression model. Two-tailed tests. The dependent variable is perception of solidarity (averaged across three solidarity questions); possible values ranged from 1 to 7. MJ is the reference category. The Control-CJ comparison did not differ ($p = .21$).

Table S3. Comparison of Public and Private Material Sanctions Conditions, Contributions, Sanctions Received, and Earnings in PGD

	Model 1: Contribution, Round 1		Model 2: Contributions, Rounds 2-9		Model 3: Average Sanctions Received		Model 4: Earnings	
	Estimate	S.E.	Estimate	S.E.	Estimate	S.E.	Estimate	S.E.
Intercept	11.33***	.99	11.23***	1.55	.03	.20	29.97***	2.52
MS_{Public}	-1.71	1.40	-.45	2.19	-.15	.29	-2.65	3.57
	$N_{observations\ (groups)}$= 104 (26)		$N_{observations\ (groups)}$= 832 (26)		$N_{observations\ (groups)}$= 936 (26)		$N_{observations\ (groups)}$= 936 (26)	

+ *p* < .10, * *p* < .05, ** *p* < .01, *** *p* < .001

Note. Two-level (Model 1) and three-level (Models 2-4) regression models. The reference category is the Private MS condition.

Table S4. Comparison of Public and Private Material Sanctions Conditions, Post-study Measures

	Model 5: Solidarity		Model 6: Contribution, One-shot PGD		Model 7: Trust		Model 8: Trustworthiness		Model 9: Generosity	
	Estimate	S.E.	Estimate	S.E.	Estimate	S.E.	Estimate	S.E.	Estimate	S.E.
Intercept	3.94***	.32	10.87***	1.42	6.58***	.53	39.71***	2.67	3.88***	.35
MS_{Public}	-.24	.46	-3.17	2.01	-.27	.75	1.37	3.76	-.35	.50

+ $p < .10$, * $p < .05$, ** $p < .01$, *** $p < .001$

Note. Two-level regression models. For all models, N = 104 participants (26 groups). The reference category is the Private MS condition.

Table S5: Contributions to the Public Good (Main Study)

	Model 1: Contributions, Round 1		Model 2: Contributions, Rounds 2-9	
	Estimate	S.E.	Estimate	S.E.
Intercept	8.25***	1.03	5.19**	1.41
MJ	4.02*	1.41	7.75***	1.93
MS	2.22+	1.26	5.81**	1.73
	$N_{observations\ (groups)}$= 216 (54)		$N_{observations\ (groups)}$= 1728 (54)	

+ $p < .10$, * $p < .05$, ** $p < .01$, *** $p < .001$

Note. Two-level (Model 1) and three-level (Model 2) regression models. Two-tailed tests. The dependent variable is contribution in round 1 for Model 1 and contribution in rounds 2 through 9 for Model 2; possible values range from 0 to 20. Control is the reference category. We also compared the MJ and MS conditions to each other, by changing the reference category to MS. For both models, MJ and MS groups did not significantly differ (p = .14 and p = .24, respectively).

Table S6: Condition and Contribution Predict Judgments/Sanctions Received (Main Study)

	Average Judgment/Sanction Received	
	Estimate	S.E.
Intercept	-.88***	.15
MJ	-.53*	.25
Contribution	.08***	.01
MJ*Contribution	.13***	.01
	$N_{observations\ (groups)}$= 1476 (41)	

+ $p < .10$, * $p < .05$, ** $p < .01$, *** $p < .001$

Note. Three-level regression model. Two-tailed tests. The dependent variable is the average judgment or sanctions ego received from ego's three other group members. Possible values ranged from -3 to 3. MS is the reference category.

Table S7. Judgments/Sanctions Received Predict Subsequent Contributions (Main Study)

	Contribution at time t	
	Estimate	S.E.
Intercept	6.09***	.99
MJ	.26	1.06
Contribution, time t-1	-.01	.05
Group Average Contribution, time t-1	.46***	.08
Group Average Sanctions/Judgments, time t-1	1.72**	.53
Average Sanction/Judgment Sent, time t-1	-.49+	.26
Average Sanction/Judgment Received, time t-1	-.62*	.26
	$N_{observations\ (groups)}$= 896 (28)	

+ $p < .10$, * $p < .05$, ** $p < .01$, *** $p < .001$

Note. Three-level regression model. Two-tailed tests. The dependent variable is contribution at time t. Possible values ranged from 0 to 20. MS is the reference category.

Table S8. Earnings (Main Study)

	Earnings	
	Estimate	S.E.
Intercept	32.86***	1.82
Control	-7.33**	2.68
MS	-4.19+	2.28
	$N_{observations\ (groups)}$= 1943 (54)	

+ $p < .10$, * $p < .05$, ** $p < .01$, *** $p < .001$

Note. Three-level regression model. Two-tailed tests. The dependent variable is earnings in a given round. We omitted one case, where a participant received negative earnings for the round, from analyses. (However, results are virtually identical when this case was included: B = -4.21 and S.E. = 2.29); possible values then ranged from 0 to 64. MJ is the reference category. We also compared the Control and MS conditions to each other, by changing the reference category to MS. MS and Control did not differ in earnings, $p = .19$.

Table S9. Retaliation and Reciprocation of Judgments and Sanctions (Main Study)

	Model 1: Negative Sanctions/Judgments Received from Alter		Model 2: Positive Sanctions/Judgments Received from Alter	
	Estimate	S.E.	Estimate	S.E.
Intercept	.88***	.09	.17	.14
MS	-.07	.09	-.72***	.19
Contribution	-.07***	.00	.08***	.00
Alter's Contribution	.02***	.00	-.00	.00
Negative Sanctions/Judgments Received from Alter, time t-1	.20***	.02		
Positive Sanctions/Judgments Received from Alter, time t-1			.19***	.02
Negative Sanctions/Judgments Sent to Alter, time t-1	.02	.03		
Positive Sanctions/Judgments Sent to Alter, time t-1			.08***	.02
MS*Negative Sanctions/Judgments Sent to Alter, time t-1	.18***	.03		
MS*Positive Sanctions/Judgments Sent to Alter, time t-1			.08*	.04
	$N_{\text{observations (groups)}}$= 2688 (28)		$N_{\text{observations (groups)}}$= 2688 (28)	

+ $p < .10$, * $p < .05$, ** $p < .01$, *** $p < .001$

Note. Four-level regression model. Two-tailed tests. Dependent variables are negative monetary sanctions or moral judgments received from a given alter (Model 1) and positive sanctions or judgments received from a given alter (Model 2). MJ is the reference category.

Table S10. Perceived Solidarity

	Average Solidarity Rating	
	Estimate	S.E.
Intercept	3.79***	.28
Control	-1.13**	.43
MS	.02	.36
	$N_{observations\ (groups)}$= 212 (53)	

+ p < .10, * p < .05, ** p < .01, *** p < .001

Note. Two-level regression model. Two-tailed tests. One group (in the Control condition) was not included in analyses because, due to a computer problem, they did not complete the post-study questionnaire. The dependent variable is perception of solidarity (averaged across three solidarity questions); possible values ranged from 1 to 7. MJ is the reference category. We also compared the Control and MS conditions to each other, by changing the reference category to MS. MS also differed significantly from control, $p < .01$.

Table S11. Post-PGD Behavioral Measures of Prosociality

	Model 1: Contribution, One-shot PGD		Model 2: Generosity		Model 3: Trust		Model 4: Trustworthiness	
	Estimate	S.E.	Estimate	S.E.	Estimate	S.E.	Estimate	S.E.
Intercept	10.85***	1.38	4.55***	.32	7.42***	.44	47.32***	2.44
Control	-4.89*	2.03	-1.34**	.48	-2.10**	.66	-9.92**	3.65
MS	-1.57	1.73	-.84*	.41	-.97+	.56	-6.92*	3.06
	$N_{\text{observations (groups)}}$= 216 (54)		$N_{\text{observations (groups)}}$= 212 (53)		$N_{\text{observations (groups)}}$= 212 (53)		$N_{\text{observations (groups)}}$= 212 (53)	

+ $p < .10$, * $p < .05$, ** $p < .01$, *** $p < .001$

Note. Two-level regression models. Two-tailed tests. One group (in the Control condition) was not included in analyses for Models 2-4 because, due to a computer problem, they did not complete these measures. Dependent variables are contributions in the one-shot PGD (possible values ranged from 0-20), generosity (possible values ranged from 0-10), trust (possible values ranged from 0-10), and trustworthiness (0-100, expressed as the percentage of the unknown endowment returned) for Models 1-4 respectively. MJ is the reference category. For Models 1 and 3, the MS-Control comparison was marginally significant (*p*s < .10); those in MS contributed and trusted more than those in the Control. For Models 2 and 4, the MS-Control comparison was not significant ($p = .26$ and $p = .37$ respectively).

Figure S1: Pilot Experiment Contributions, by Condition and Round

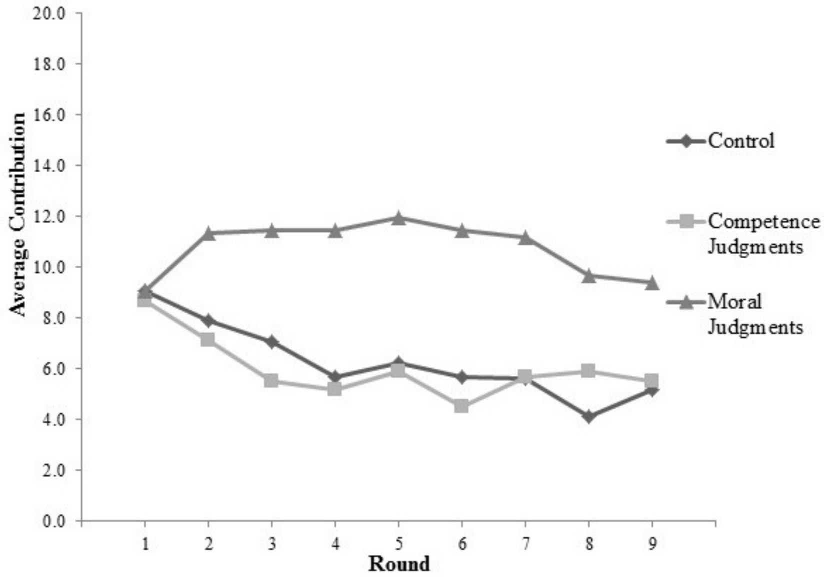

Full Text of Study Materials

All materials for both studies were presented via computer, and were programmed in zTree version 3.3.12 (Fischbacher 2007).

Pilot Study

[Instructions Screen 1]

In today's study, you will take part in a series of "investment decisions." Your earnings in today's study will partly depend on how well you understand the instructions. So please read all instructions and examples carefully.

The basic directions are as follows: you will be completing this study in a group of four. Your group members are three other participants currently in the lab. You will not meet your group members at any time, nor will you learn any identifying information about your group members. Likewise, your group members will not learn any identifying information about you.

The study consists of several periods (or rounds). At the start of each round, you (and each of your other group members) get 20 points. You can contribute anywhere from 0 to 20 of these points to a "group fund." Any points you do not contribute to the group fund remain in your personal fund, for you to keep.

Anything that is contributed to the group fund will be doubled. Then, the doubled amount will be divided between all four of the members of your group, whether or not they contributed to the group fund. (Similarly, other group members' contributions to the group fund will be doubled and redistributed equally among all members of the group.) Your total earnings per round are your share of the earnings from the group fund, plus whatever you did not invest. The same goes for other group members.

Points earned over each round will be translated into dollars at the end of the study. **So, the more points you earn, the more money you will receive.**

Make sure you have carefully read and understand the instructions. Next, you will read over a few examples. If you have any questions about the instructions, you may slightly open your door and a research assistant will be with you in a moment.

If you understand the instructions, click "Continue."

[Example Screen 1]

Let's go over an example.

Imagine that each group member invests all 20 of their points. Thus, there are now 80 points in the group fund (20 points from each of 4 group members).

Since anything in the group fund is doubled, the 80 points become 160 points. Then, the group fund is divided by four (for each of the four group members). So, each group member receives 40 points.

Since everyone invested all of their points to the group fund, each player finishes the period with 40 points (40 earned from the group fund + 0 kept in the personal fund).

If you have any questions about this example, please slightly open your door and a research assistant will be with you in a moment.

If you understand the example, click "Continue."

[Example Screen 2]

Here's another example.

Imagine that each group member invests none of their 20 points. Now, there are zero points in the group fund.

Since there are no points in the group fund to double, and no points to divide by all the group members, everyone earns 20 points from this round: 0 points from the group fund + 20 points kept in the personal fund.

If you have any questions, please slightly open your door and a research assistant will be with you in a moment.

If you understand the example, click "Continue."

[Example Screen 3]

Imagine that three group members invest all 20 of their points, and one group member invests none of their points. Thus, there are 60 points in the group fund (20 points x 3 group members).

The 60 points in the group fund gets doubled to 120 points. Then, the 120 points gets divided into four so that every group member gets 30 points.

The three group members who invested their points end the round with 30 points each (30 points from the group fund + 0 points kept in the personal fund). The group member who invested nothing ends the round with 50 points (30 earned from the group fund + 20 kept in the personal fund).

If you have any questions, please slightly open your door and a research assistant will be with you in a moment.

If you understand the example, click "Continue."

Participants then completed a series of quiz questions (available upon request) before proceeding with the following instructions.

[Instructions Screen 2—Competence Judgments and Moral Judgments conditions; Control condition proceeded to Instructions Screen 3]

One more thing before we begin the investment decisions: in between each round of the task, you will answer several questions on your perceptions of the other participants. Similarly, the other participants will answer questions about their perceptions of you.

Specifically, after each round, you will see each participant's ID number and decision for that round. Below each ID number and decision, you will be asked to fill in a number ranking each participant, on a scale of one to seven, for several different questions.

For example, if the question asked you to rank how "happy" the participant was-- on a scale of one to seven, where one equals "very unhappy" and seven equals "very happy"-- you would fill in a number, from one to seven, in the corresponding box below each participant's identification number.

After each group member answers each question, everyone will be able to see each others' ratings. Click "Continue" to view an example.

[Judgments Display Screen Example—Competence Judgments and Moral Judgments conditions]

This is an example of the screen you will see after each group member has rated everyone in the group. Your Subject ID will be clearly displayed here so you can see your group members' ratings of you. For example, if your Subject ID was 1, you would find the ratings others made of you by looking at the "Ratings of Subject 1" box. Similarly, your group members will be able to see your ratings (and others' ratings) of them. (In the example, ratings were about others' happiness on a scale of 1 to 7. We may ask different questions, and the questions and scale will be clearly displayed here, as well.)

Ratings of Subject 1:	
Rating Made by Subject:	Happy Rating:
2	6
3	5

Ratings of Subject 2:	
Rating Made by Subject:	Happy Rating:
1	6
3	4

Ratings of Subject 3:	
Rating Made By Subject:	Happy Rating:
1	5
2	5

Ratings of Subject 4:	
Rating Made by Subject:	Happy Rating:
1	3
2	2

4	7	4	4	4	4	3	2

Be sure you have read the instructions carefully. You will answer one more quiz question before we move on.

Again, participants were quizzed to ensure understanding before proceeding with instructions.

[Instructions Screen 3]

We will now move on to the "investment decisions" portion of the study. Click "Continue."

Once all group members have indicated that they are ready, we will begin.

After the PGD was completed, participants answered three post-study questions to measure perceived group solidarity:

1. How much solidarity do you think the group had?
2. How much do you think the group felt like a team?
3. How much do you think the group stuck together?

Possible response categories ranged from 1 (Not at all) to 7 (A great deal).

Main Study Materials

Participants in the main study first saw Instructions Screen 1, and Example Screens 1-4 exactly as they were presented in the pilot study. (They also received similar quiz questions.) They then saw the instructions given here.

[Instructions Screen 2—Moral Judgments condition only]

One more thing before we begin the investment decisions task: after each round of the task, after you see each group member's investment decision, you will have the opportunity to **make judgments** about your group members' behaviors.

Similarly, the other participants will be able to make judgments about your behavior, and about each other's behaviors.

Specifically, after each round, you will see each participant's ID number and decision for that round. Below each ID number and decision, you may indicate the extent to which you believe the group member's behavior was **immoral** or **moral** (on a scale of -3 to 3).

After each person indicates the immorality or morality of other group members' behaviors (if they choose to do so), all group members will be able to see the results. That is, the screen will display who made judgments, and to whom. Then, all group members

will see the sum of ratings they received from others (which can range from -9, or very immoral, to 9, or very moral), and the sum of ratings they made of others.

Please make sure that you have read the instructions carefully. If you have any questions, slightly open your door and a research assistant will be with you in a moment. Otherwise, click "Continue."

[Instructions Screen 2—Public [Private] Material Sanctions conditions only]

One more thing before we begin the investment decisions task: after each round of the task, after you see each group member's investment decision, you will have the opportunity to spend points to **add to or deduct from** any group member's earnings.

Similarly, the other participants will be able to spend points to make deductions from or additions to your earnings, and to/from each other's earnings.

Specifically, after each round, you will see each participant's ID number and decision for that round. Below each ID number and decision, you may indicate if you wish to **deduct points from** or **add points to** each other group member's earnings. **Each point** you spend will reduce (if you choose to reduce points) or increase (if you choose to add points) that group member's earnings by **three points.** You may spend up to 3 points to deduct from or add to any group member's earnings. Therefore, the maximum amount you can add to or deduct from a given group member is nine points.

After each person makes deductions from or additions to other group members' earnings (if they choose to do so), all group members will be able to see the results [each group member will be able to see only his/her results]. That is, the screen will display who made deductions or additions, and to whom. Then, all group members will see the sum of earnings changes they received from others (which can range from -9, or nine points deducted, to 9, or nine points added), and the sum of the deductions or additions they made to others. [That is, the screen will display how many deductions or additions were made to your earnings, but not deductions or additions made to the others', or which group member made them.]

Please make sure that you have read the instructions carefully. If you have any questions, slightly open your door and a research assistant will be with you in a moment. Otherwise, click "Continue."

[Instructions Screen 3—all conditions]

We will now move on to the investment decisions portion of the study. Click "Continue."

Once all group members have indicated that they are ready, we will begin.

After the PGD was completed, participants completed several post-study measures. We reproduce these below, in the order in which they were presented.

One-shot version of the PGD

[Instructions for MJ and MS in brackets; (MJ in parentheses)]

> For the next decision task of today's study, you and your group members will again begin 20 points and can contribute any portion of these points to the group fund. Any points you do not contribute to the group fund remain in your personal fund, for you to keep, and anything contributed to the group fund will be **doubled** and divided between all four of the members of your group.
>
> Unlike the last task, you will make this decision exactly **once** and you will **not** receive **any** feedback on others' decisions and earnings for this task. That is, you won't see any information about what the others contributed, or how much you earned, unlike you did in the previous rounds. [Relatedly, since you will not see others' decisions or earnings, **you will not have the opportunity to add or deduct earnings from (make judgments about) others-- nor will they be able to add or deduct from (make judgments about) you.]** Your earnings from this task will be added to your earnings from the previous task, which will be paid to you at the end of today's study.

Perceptions of Solidarity

We used the same solidarity measure presented in the Pilot Study; see previous section.

Trust Dilemma

Participants next completed a standard trust dilemma (Berg et al. 1995) as both the Truster (in the instructions, the “Sender”) and Trustee (the “Returner”). They read the instructions for both roles of the task, answered several quiz questions, made their decision as Sender, and then made their decision as Returner. In both decisions, participants were given the ID number of the group number with whom they were paired; they were never paired with the same other twice (i.e., for both decisions). Finally, when making their decisions in the Returner role, participants did not know how much the Sender had chosen to send—rather, they were asked to indicate what percentage of the (unknown) endowment they would return. See instructions below.

[Instructions Screen 1]

> For the next task of today's study, you will take part in a different scenario with your group members. Your earnings in this scenario will be added to your earnings from the previous task.
>
> In this scenario, there are two roles: Sender and Returner. You will be assigned to one role, and one of your group members will be assigned to the other. Later, you will be assigned to the other role and paired with a different group member. We will always let you know the ID number of the group member with whom you have been paired before you make your decision in the task.

Your earnings from this scenario will be either your earnings as Sender or your earnings as Returner. The role for which you will be paid will be randomly determined at the end of the study. Therefore, it is important that you understand BOTH roles, which are described on the next page.

Click Continue.

[Instructions Screen 2]

The basic instructions for this scenario are as follows: the Sender is given 10 points. The Sender then decides how many of the 10 points (if any) to send to the Returner, and how many to keep for him/herself. If the Sender sends zero, the Sender will end the task with ten points and the Returner will end with zero points. If the Sender sends some amount greater than zero, the sent amount will be TRIPLED. The Returner will decide what percentage of the tripled amount-- from 0% to 100%-- to return to the Sender.

Again, the Sender can send any amount from 0 to 10, and the Returner can return any percentage of the tripled investment, from 0% up to the entire tripled investment (100%).

For example, if the Sender chooses to send 10, that amount is tripled to become 30 points. If the Returner decides to return 50% of the endowment to the Sender, the Returner and the Sender will end with 15 points each. If the Returner decides to return 0% of the endowment to the Sender, the Sender will end with 0 points and the Returner will end with 30 points. And so on.

Finally: although you WILL know the ID number of the group member you are paired with for each task before making a decision, you will NOT receive any feedback about others' decisions in the task until the end of the study. That is, Returner will NOT be told the exact amount of the endowment they were sent by the Sender. They will simply indicate the percentage of the (unknown) endowment they will return to the Sender. Likewise, Senders will not be told the proportion of the endowment the Returner chose to return.

You will participate in both roles, so make sure you have read the instructions carefully. Next, you will answer several quiz questions to ensure your understanding.

For this and other behavioral measures, participants completed a series of quiz questions designed to ensure that they understood the instructions (available upon request from the authors).

[Instructions as Sender]

You have been assigned to the role of SENDER.

That is, you now have an endowment of 10 and can decide how much to send to the Returner. The Returner is one of your fellow group members-- we will tell you the group member's ID number in a moment. Likewise, the person with whom you are paired will be given your ID number.

[Input as Sender]

You are paired with Participant [X].

How much of your ten point endowment would you like to send to Participant [X]?

Enter a number from 0 to 10.

[Instructions as Receiver]

Now, as described earlier, you will change to the RETURNER role. Like before, you will make your decision with one of your group members (NOT the group member you were paired with in the previous decision).

Remember: as the Returner, you will be paired with a Sender, who has an endowment of 10 and has chosen how much, if any, to send to you. This amount (if any) has been tripled. As before, we will not give you any feedback about other participants' decisions until the end of the study-- that is, **we will not tell you the exact amount that you were sent by the Sender with whom you have been paired.**

Instead, you will indicate the **percentage** of the endowment that you would like to return to the Sender, regardless of how much s/he sent you. For instance, you may indicate that you wish to return 50% of the tripled amount to Sender X, 100% of the tripled amount to Sender Y, or 0% of the tripled amount to Sender Z. You may choose any percentage to return to the Sender-- from 0% to 100% of the endowment.

The Sender is another one of your fellow group members-- we will tell you the Sender's ID number before you make your decision. Likewise, the Sender will know your ID number before s/he makes a decision. However, you will not receive any feedback about your or your group members' decisions until the end of the study.

Click "Continue" to begin.

[Input as Returner]

You are paired with Participant [X].

What percentage of the endowment sent by Participant [X] would you like to return to Participant [X]?

Enter a number from 0 to 100.

Dictator Game

Participants next completed a standard measure of generosity, the Dictator Game (cite?). Like in the Trust Game, they were told the ID number of the group member with whom they were paired. Participants were always paired with the one other group member with whom they had not already been paired as either Sender or Returner in the Trust Game.

[Instructions, Screen 1]

> For the next task of today's study, you will take part in a different scenario. Your earnings in this scenario will be added to your earnings from the previous task.
>
> Click Continue.

[Instructions, Screen 2]

> In this scenario, there are two roles: Decider and Receiver. You will be assigned to one role, and one of your group members will be assigned to the other. Later, you will be assigned to the other role and paired another group member. We will always let you know the ID number of the group member with whom you have been paired before you make your decision in the task.
>
> Your earnings from this scenario will be either your earnings as Decider (paired with one group member) or your earnings as Receiver (paired with one group member). The role for which you will be paid will be randomly determined at the end of the study.
>
> The basic instructions for this scenario are as follows: the Decider is given 10 points. The Decider's task is to decide how many of the 10 points (if any) to send to the Receiver and how many to keep for him/herself.
>
> Any amount the Decider does not send is his or hers to keep. Likewise, any amount the Decider sends to the Receiver is the Receiver's to keep. For instance, if the Decider sends five points and keeps five points, then the Decider and the Receiver each end with five points. If the Decider sends zero points and keeps ten points, the Decider ends with ten points and the Receiver gets nothing. And so on.
>
> Make sure you have read the instructions carefully. Next, you will answer several quiz questions to ensure your understanding

[Decider Instructions Screen]

> You will begin the task in the role of DECIDER.

That is, you now have an endowment of 10 and can decide how much to send to the Receiver. The Receiver is one of your fellow group members-- we will tell you the group member's ID number in a moment.

[Decider Input Screen]

You are paired with Participant [X].

How much of your ten point endowment would you like to send to Participant [X]?

Enter a number from 0 to 10.

References

1 Goodwin, G. P., Piazza, J. & Rozin, P. Moral character predominates in person perception and evaluation. *J Pers Soc Psychol* **106**, 148-168, doi:10.1037/a0034726 (2014).

2 Fiske, S. T., Cuddy, A. J. & Glick, P. Universal dimensions of social cognition: warmth and competence. *Trends in cognitive sciences* **11**, 77-83, doi:10.1016/j.tics.2006.11.005 (2007).

3 Fischbacher, U. z-Tree: Zurich toolbox for ready-made economic experiments. *Experimental economics* **10**, 171-178 (2007).

4 Baldassarri, D. & Grossman, G. Centralized sanctioning and legitimate authority promote cooperation in humans. *Proceedings of the National Academy of Sciences of the United States of America* **108**, 11023-11027, doi:10.1073/pnas.1105456108 (2011).

5 Fehr, E. & Gächter, S. Altruistic punishment in humans. *Nature* **415**, 137-140 (2002).

6 Peeters, R. & Vorsatz, M. Immaterial rewards and sanctions in a voluntary contribution experiment. *Economic Inquiry* **51**, 1442-1456 (2013).

7 Willer, R. Groups reward individual sacrifice: The status solution to the collective action problem. *American Sociological Review* **74**, 23-43 (2009).

8 Ostrom, E. Collective action and the evolution of social norms. *The Journal of Economic Perspectives*, 137-158 (2000).

9 Sell, J. & Wilson, R. K. Levels of information and contributions to public goods. *Social Forces* **70**, 107-124 (1991).
